# Mesenchymal and Vascular Dissemination Markers, Erythroblastosis Virus E26 Oncogene Homolog (ERG) and Alpha Smooth Muscle Actin (α-SMA), in Colorectal Cancer and Adjacent Tissue, Pericytes or Microvascular Density

**DOI:** 10.7759/cureus.50059

**Published:** 2023-12-06

**Authors:** Jiyan Haji, Mayada Ilias

**Affiliations:** 1 Department of Biology, College of Science, University of Duhok, Duhok, IRQ; 2 Department of Pathology, College of Medicine, University of Duhok, Duhok, IRQ

**Keywords:** microvascular density, pericytes, adjacent tissue, colorectal cancer, sma, erg

## Abstract

The harmony between malignant cells and the adjacent microenvironment is a sophisticated subject; however, it seems to play an important role in cancer evolution. This study aimed to assess the microvascular density (MVD) and the mean pericyte number in the tumor and adjacent tissue, and to correlate the results with special histopathological prognostic variables of the tumor. The study included 48 colorectal cancer (CRC) cases diagnosed in the central lab of Duhok. The immunohistochemical (IHC) expressions of the mesenchymal and vascular dissemination markers, erythroblastosis virus E26 oncogene homolog (ERG, a member of the ETS family of transcription factors) and alpha smooth muscle actin (α-SMA) for microvascular density and pericytes, were assessed in tumor cells and in adjacent tissue around the tumor and then correlated to clinicopathological variables with a special concentration on inflammatory reaction, tumor budding, tumor deposition, and lymphovascular invasion. The results showed that the MVD was significantly higher outside the tumor in T1 and T2 compared with T3 and T4. Moreover, it was significantly higher in grade I when compared to grades II and III within the tumor. However, no correlation was found between the MVD and the special histopathological variables that had been studied. On the other hand, the low mean pericyte showed multiple significant associations outside tumor areas, with special histopathological features including a severe inflammatory reaction, a positive tumor deposit, and a negative lymphovascular invasion. These findings may indicate that defective or transformed pericytes around the tumor can participate in the development of the tumor and, subsequently, the outcome and prognosis.

## Introduction

In the last two decades the microenvironment of all human cancers, including colorectal cancer (CRC), has become extensively connected with carcinogenesis. It has been demonstrated that several components within the tumor microenvironment (TME) could affect the behavior and spread of malignancy, for instance, promoting and enabling cell proliferation of nonmalignant cells in the tumor microenvironment play crucial roles in all stages of cancer disease, furthermore, the presence of tumor cells that interact with surrounding cells via the lymphatic and circulatory systems affect the development and progression of cancer, in which the tumor cells penetrate the blood vessel wall, and travel with the bloodstream to form a metastasis at a distant location [1-3]. The TME is made up of a constantly changing combination of extracellular factors that surround cancer cells and could play structural and functional roles in both physiological and pathological conditions. Besides malignant cells, the TME contains a variety of other types of cells, such as adipocytes, fibroblasts, tumor vasculature, lymphocytes, dendritic cells, and cancer-associated fibroblasts. The distinct immune capacities of each of these cell types determine if the tumor will persist and impact nearby cells; therefore, it has become increasingly clear how tumor growth can be impacted by components that make up the TME and not only by genetic or epigenetic modifications of cancer cells [2,3]. The formation of new blood vessels is stimulated by angiogenic factors secreted by tumor cells and other cells within the TME. This process enables the tumor to grow to a biologically detectable size and creates a channel for tumor cells to spread. These recently created blood vessels in cancer have very varied features; they are frequently leaky, condensed, and exhibit uneven branching patterns, which results in variable blood flow and nutrient supply to the tumor [4,5]. The blood vessels within and adjacent tissue of the tumor have been identified as a potential target for cancer therapy. Endothelial cells, pericytes, endothelial progenitor cells, smooth muscle cells, and extracellular matrix make up the blood vascular component of the tumor and its adjacent tissue [6]. In addition to acting as a gateway for the passage of nutrients and oxygen, as well as for the elimination of metabolic waste, the blood vessels in tumor tissues enable the metastasis of tumor cells. On the contrary, insufficient blood supply may prevent tumor growth, even cause tumor diminishment, and even result in dying cancer cells [6,7]. Additionally, the stem/serrated/mesenchymal transcriptional subtype of CRC, which had a poor prognosis, exhibits high expression of cancer-associated fibroblasts, leukocytes, and endothelial cells. Furthermore, endothelial cells strongly promote Notch signaling and cancer stem cells in CRC. These endothelial cells in capillaries are wrapped up by pericytes, which are mural cells that are immersed in the same basement membrane [8]. The preservation of tumor neovasculature depends on pericytes; their decreasing coverage of tumor microvessels may compromise vascular integrity, resulting in metastasis [9,10]. It has been shown that CRC stem cells are capable of creating pericytes that facilitate tumor development and vascular function [11].

The number of tiny vessels per square millimeter of tumor area is represented as microvascular density (MVD), which represents a quantitative indicator of the degree of angiogenesis inside neoplastic tissue. Measuring MVD is considered a standard method for the evaluation of tumor angiogenesis, moreover, the level of tumor angiogenesis based on MVD could potentially be valuable as a prognostic biomarker of tumors [12-14]. Multiple endothelial markers of immunohistochemistry (IHC) can be used to recognize the recently developed small blood vessels and differentiate them from the existing blood vessels [12-15]. Among the commonly used IHC markers are CD31, CD34, alpha smooth muscle actin (α-SMA), and erythroblastosis virus E26 oncogene homolog (ERG). ERG is an avian-vets member of the ETS family of transcription factors that plays a role in tumor angiogenesis and is effectively expressed in vascular endothelial cells, not stromal or tumor cells. Conversely, CD31 and CD34 exhibited weak to moderate reactivity [15]. Moreover, it has been demonstrated that α-SMA has effective expression in vessels and pericytes [12]. This study aimed to estimate the MVD in tumor and adjacent tissue of CRC patients by using two IHC markers, ERG and α-SMA, the first one to stain the endothelial cells, and the second to stain the pericytes; to assess both the MVD and the mean pericytes number, then to correlate the results with general clinicopathological variables and special histopathological changes including inflammatory reaction, tumor budding, tumor deposition and lymphovascular invasion.

## Materials and methods

Patients and methods

This cross-sectional study included 48 CRC patients selected between January 1, 2013 to December 31, 2015. Samples were obtained as formalin-fixed, paraffin-embedded (FFPE) blocks from the central lab, Duhok government. The approval of the study was done via a committee chosen by the College of Science/Biology Department/University of Duhok and the Medical Ethics Committee of the General Directorate of Health in Duhok. The associated pathology sheets for each patient were utilized to access the clinical information, such as the patient's age, gender, phone number, and the name of the operating surgeon. Sections taken from the FFPE blocks were re-stained with hematoxylin and eosin (H&E) for reexamination to confirm the diagnosis and other findings in the report and to focus on the special histopathological features of the tumor, including: an inflammatory reaction (mild, moderate, or severe) according to the number of inflammatory cells; tumor budding (high; ≥10; intermediate; 5-9; low; 0-4) represents the number of cancerous cells that have separated from the invasive front of the tumor; tumor deposit (negative, positive); and lymphovascular invasion (negative, positive). These features have been assessed depending on the guidelines of the American Joint Committee on Cancer of the CRC [16]. This re-examination has been carried out by two specialists in pathology. The present study included and excluded cases depending on certain criteria, as shown in Table 1.

**Table 1:** Inclusion and exclusion criteria of the cases of the study. CRC: colorectal cancer, LNs: lymph nodes.

Inclusion criteria	Exclusion criteria
Patient with biopsy confirmed to be CRC	Colonoscopy samples
The biopsy should include the tumor and part of the colon with safe margins and removal of at least 12 lymph nodes (LNs)	Biopsy without safe margins or with less than 12 LNs involvement
Complete data in the pathology reports (for example, age, sex, site, and gross appearance)	

The cases were categorized according to the World Health Organization's (WHO's) histologic categorization of CRC [17], both grading and histological staging for the degree of tumor invasion (T) and positive lymph node (LN) involvement (N) “as the histological part of the TNM staging system” were carried out based to the guidance of the American Joint Committee on Cancer of the CRC [16].

Immunohistochemistry analysis

Avidin-biotin complex (ABC) method of staining was performed manually. During the re-examination of the H&E slides, special attention was paid to selecting the most suitable areas for the tumor primary site and the adjacent uninvolved area. Then 4 μm thick sections were obtained from each block and mounted on a positive charge slide (Dako, Glostrup, Denmark) for the staining procedure. Slides were heated in an oven for one hour at 60°C, followed by deparaffinization in xylene and rehydration in a series of ethanol solutions. Pre staining step of antigen retrieval was performed for all samples, depending on the Dako PT Link instrument. After that the slides were washed with phosphate buffer saline (PBS), endogenous peroxidase in tissue samples was blocked by adding 3% hydrogen peroxidase (H2O2) on tissue sections on the slides for five minutes, then slides were rinsed with PBS, after that incubated with ready-to-use primary antibody at room temperature from 20-30 minutes, rinsed again, then they were exposed to secondary antibody for 20 minutes. The slides were rinsed twice with PBS for five minutes. The antigen-antibody complex was visualized by staining the sections on slides for 10 minutes with diaminobenzidine/hydrogen peroxidase chromogen solution, then rinsed again with PBS. After that they were counterstained with hematoxylin for five minutes, then rinsed three times; first with distilled water then with PBS and after that with distilled water again. Finally, all slides were dehydrated with a series of solutions of ethanol. Positive and negative controls were used for each marker.

The primary antibodies used were monoclonal rabbit anti-human ERG, clone EP111 and monoclonal mouse anti-human α-smooth muscle actin, clone 1A4 (both from Dako).

Staining pattern

A separated double IHC staining was carried out to evaluate the MVD depending on the endothelial cells (confirmed by the ERG) and also the pericyte (confirmed by the α-SMA). Then the slides of α-SMA were examined to calculate the number of pericytes around the microvessels in both the tumor area and the adjacent unaffected area. The pericytes were assessed by counting the microvascular pericytes positive for α-SMA over the total number of microvessels. The average percentage of three hot spots was then calculated to get the mean pericyte number.

MVD estimation

Clusters of pericytes, confirmed by α-SMA, or endothelial cells, confirmed by ERG, around blood cells or empty space were identified as a microvessel. However, any vessel surrounded by muscular layers was not counted. At the beginning of the examination, the slides were examined by an Olympus microscope, Tokyo, Japan, at the magnification of 4× to select three areas of the highest concentration of microvessel (hot spots). Then, at a magnification of 10× in each one of these histological areas, the number of microvessels was counted. The MVD then was assessed by the number of microvessels/mm2 according to Svagzdys [16]. The calculation was carried out in the tumor and in areas free of malignancy (separated by at least one histological field at 10×). This type of IHC staining can confirm the spaces are retraction artifacts or vessels by the detection of positive endothelial cells or pericytes or both in newly formed vessels.

The IHC slides were read by the pathologist blindly, without knowing the other demographic and clinical features. The staining pattern of ERG was nuclear staining to identify endothelial cells of blood and lymphatic vessels. The staining pattern of α-SMA was cytoplasmic or membranous, assessing the extent of the pericytes surrounding the blood vessels continuously or in a discontinuous pattern. Pericytes were considered present if α-SMA was detected anywhere along the vessel as a single layer of α-SMA-positive cells.

Statistical analysis

The comparisons of ERG and α-SMA expression of patients with CRC with different characteristics were examined in an independent t-test or ANOVA one-way. The pairwise comparisons were examined using a Tukey test. The level of difference was determined in a P<0.05. The statistical calculations were performed using the statistical software JM Pro 14.3.0 (https://www.jmp.com/en_us/home.html). 

## Results

The age range of the patients included in this study was 19-100 years, with a mean of 58.40 years and a SD of 16.98. The males represented more than half of the cases (58.33%), as seen in Table 2. The highest site for CRC was seen in the rectum (37.50). The vast majority presented for the first time in grade II as “moderately differentiated” (91.67%). However, more than three-quarters were in tumor depth T3 or T4 (77.08%). Half of the CRC patients had LN involvement at the time of diagnosis. A severe inflammatory reaction was seen in 45.83% of cases. No or low tumor budding was seen in 25% of the patients, and the remaining 75% showed either intermediate or high tumor budding (29.17% and 45.83%), respectively. Tumor deposit was negative in the majority of the cases (87.50%), and 45.83% of cases showed positive lymphovascular invasion (Table 2)

**Table 2:** General and special clinicopathological features of colorectal cancer patients. T: tumor, T1: tumor invades submucosa (through the muscularis mucosa but not into the muscularis propria), T2: tumor invades muscularis propria, T3: tumor invades through the muscularis propria into the pericolorectal tissues, T4: tumor invades through the visceral peritoneum or adheres to other adjacent organs or structures

General clinicopathological features	Number	Percent
Age groups		
< 60	20	41.67
≥ 60	28	58.33
Gender		
Male	28	58.33
Female	20	41.67
Tumor location		
Left side	16	33.33
Right side	14	29.17
Rectum	18	37.50
Tumor grade		
I “Well differentiated”	2	4.17
II “Moderately differentiated”	44	91.67
III “Poorly differentiated”	2	4.17
Tumor (T)		
T1+T2	11	22.92
T3+T4	37	77.08
Positive lymph node		
Not involved	24	50.00
Involved	24	50.00
Special histopathological features		
Inflammatory reaction		
Mild	7	14.58
Moderate	19	39.58
Severe	22	45.83
Tumor budding		
Low (0-4)	12	25.00
Intermediate (5-9)	14	29.17
High (≥10)	22	45.83
Tumor deposits		
Negative	42	87.50
Positive	6	12.50
Lymphovascular invasion		
Negative	26	54.17
Positive	22	45.83

**Figure 1:**
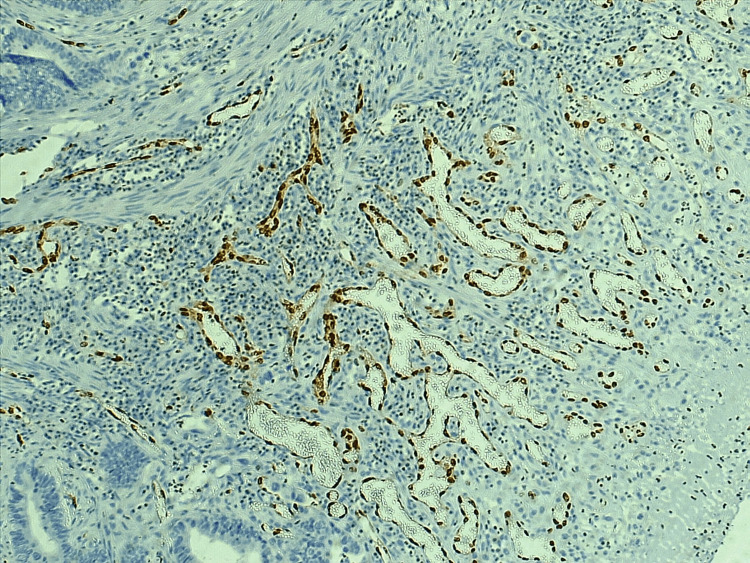
The ERG staining the nucleus of endothelial cells lining the vessels in hot spot area within tumor region, 10X. ERG: erythroblastosis virus E26 oncogene homolog

**Figure 2:**
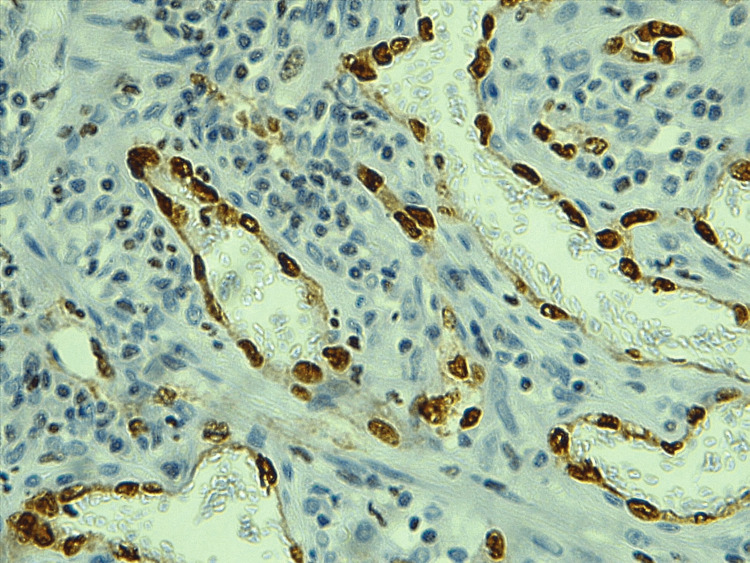
The ERG staining the nucleus of endothelial cells lining the vessels in hot spot area within tumor region, 40X ERG: erythroblastosis virus E26 oncogene homolog

**Figure 3:**
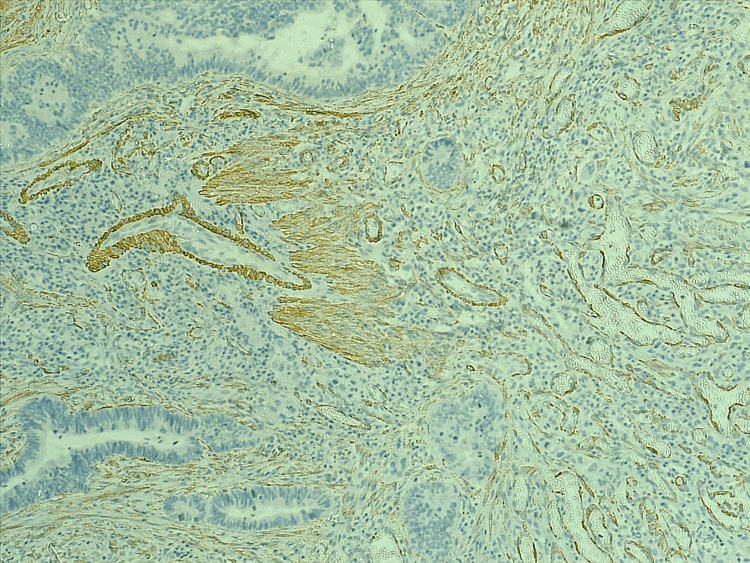
The α-SMA staining the pericytes around vessels in hot spot area within tumor region, 10X. α-SMA: alpha smooth muscle actin

**Figure 4:**
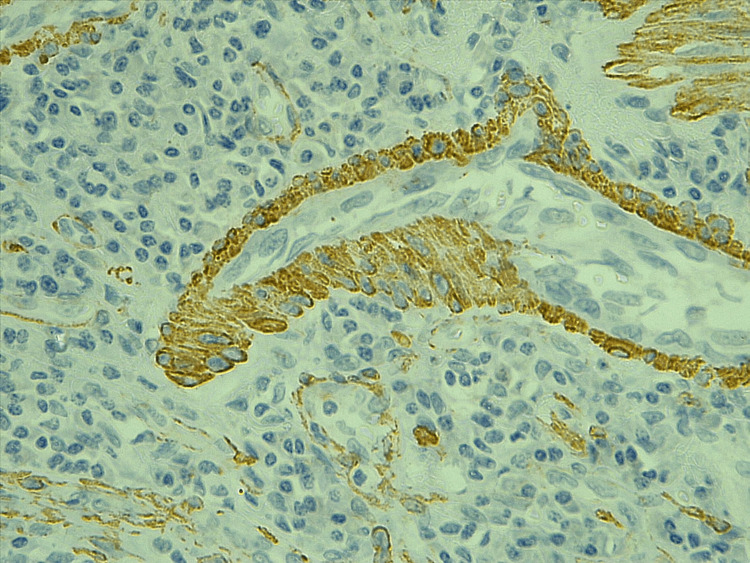
The α-SMA staining the pericytes around vessels in hot spot area within tumor region, 40X. α-SMA: alpha smooth muscle actin

MVD was estimated in tumor and adjacent tissue by using the ERG and the α-SMA, as seen in Figures 1, 2, 3, 4, the mean number of vessels per three hot areas representing the MVD (Table 3). The highest mean of microvascularity was that detected by the α-SMA in the tumor areas (29.67) with the highest maximum number of vessels, while the lowest mean of micro-vascularity was detected by the ERG in the adjacent areas of tumor (21.52) with the lowest maximum number of vessels. Oppositely, the mean number of pericytes positive for α-SMA was higher in the adjacent tissue of the tumor than in the tumor itself.

**Table 3:** MVD estimated by α-SMA and ERG and pericytes estimated by α-SMA in tumor and adjacent tissue. MVD: microvascular density, SD: standard deviation, α-SMA: alpha smooth muscle actin, ERG: erythroblastosis virus E26 oncogene homolog

Mean number of vessels per three hot areas	Mean(SD)	Minimum-Maximum
Confirmed by ERG expression in tumor	25.77(13.00)	7-96
Confirmed by ERG expression in adjacent tissue	21.52(12.33)	6-58
Confirmed by α-SMA expression in tumor	29.67(14.62)	6-100
Confirmed by α-SMA expression in adjacent tissue	22.43(9.55)	6-70
Mean number of pericytes positive for α-SMA		
In vessels of tumor	91.57(6.56)	10-100
In vessels of adjacent tissue	96.22(3.42)	84-100

The association of the MVD assessed by ERG and the general clinicopathological variables is shown in Table 4. The results show that in cancer adjacent tissue, the mean of MVD was significantly higher in the left side CRC when compared with both the rectum and the right side. The association with the tumor grade also revealed a significant correlation when we compared the MVD in well-differentiated tumors with both moderate and poor differentiation inside the tumor vascularity only (but not in the adjacent areas). The other finding was that for the cases in T3 or T4 the mean of the MVD was statistically lower outside the tumor than in T1 and T2. No correlation was detected with other variables like age, sex, and LN involvement (Table 4). 

**Table 4:** Association of the clinicopathological features with MVD by ERG expression in tumor and adjacent areas among patients with CRC. MVD: microvascular density, CRC: colorectal cancer, n: number, SD: standard deviation, T: tumor, T1: tumor invades submucosa (through the muscularis mucosa but not into the muscularis propria); T2: tumor invades muscularis propria; T3: tumor invades through the muscularis propria into the pericolorectal tissues; T4: tumor invades through the visceral peritoneum or adheres to other adjacent organs or structures, LN: lymph node; N1: metastasis in one to three regional lymph nodes; N2: metastasis in four or more regional lymph nodes. P-value <0.05 was considered significant.

Clinicopathological features (n = 48)	MVD by ERG expression			
	In Tumor Mean (SD)	P and pairwise comparisons	Adjacent areas Mean (SD)	P and pairwise comparisons
Age				
<60	25.55(11.66)	0.9232	23.30 (13.62)	0.1949
≥60	25.93(14.12)		18.93 (9.18)	
Sex				
Male	23.18(12.72)	0.2033	18.00 (8.95)	0.1282
Female	27.83(10.55)		22.55 (10.95)	
Tumor location			0.0018	
Left	23.4 (8.46)	0.2238	30.06 (13.64)	left side vs. Rectum (P=0.0031)
Right	22.93(16.18)		17.64 (11.48)	left side vs. right side (P=0.0092)
Rectum	29.94(12.95)		16.94 (7.23)	right side vs. Rectum (P=0.9825
Tumor grade		0.0019		
I “Well differentiated”	65.5 (43.13)	Well vs. Moderate (P=0.0012)	35.0 (1.41)	0.0826
II “Moderately differentiated”	25.45(13.34)	Well vs. Poor (P=0.0349)	19.45 (9.81)	
III “Poorly differentiated”	28.0 (4.24)	Moderate vs. Poor (P=0.9683)	16.0 (8.49)	
Tumor (T)				
T1+ T2	30.8 (15.48)	0.1801	28.7 (17.81)	0.0444
T3+ T4	24.37(12.47)		19.72 (10.18)	
LN involvement				
Not involved	21.05 (9.32)	0.1130	23.13 (12.38)	0.4684
Involved			Involved	
N1	25.40(12.84)		21.6 (14.74)	
N2	30.11(10.75)		17.11 (6.53)	

Special tumor histopathological variables were evaluated including; inflammation, tumor budding, tumor deposits and lymphovascular invasion, and were correlated to the mean MVD by ERG expression in tumor and adjacent areas, as seen in Table 5. No correlation was seen among the MVD and these variables. Although the MVD was slightly high when the tumor budding was intermediate and even higher when the budding was high within the tumor areas, these results were statistically not significant.

**Table 5:** Association of the special histopathological variables with MVD by ERG expression in tumor and adjacent areas among patients with CRC. MVD: microvascular density, CRC: colorectal cancer, n: number, SD: standard deviation. P-value <0.05 was considered significant.

Special histopathological variables (n = 48)	MVD by ERG expression			
	In tumor Mean (SD)	P and pairwise comparisons	Adjacent areas Mean (SD)	P and pairwise comparisons
Inflammatory reaction				
Mild	25.57 (14.39)	0.3173	16.86 (10.21)	0.1690
Moderate	20.33 (8.17)		25.53 (14.76)	
Severe	25.55 (10.91)		19.55 (9.90)	
Tumor budding				
Low	19.55 (9.86)	0.2059	23.33 (15.51)	0.5525
Intermediate	25.57 (12.62)		18.31 (8.77)	
High	27.48 (12.24)		20.86 (10.25)	
Tumor deposits				
Not present	25.66 (13.31)	0.8841	19.65 (10.17)	0.5720
Present	26.50 (11.67)		22.17 (9.52)	
Lymphovascular invasion				
Negative	23.92 (14.07)	0.3044	19.8 (10.06)	0.5441
Positive	27.86 (11.62)		21.82 (12.55)	

When the mean MVD was assessed by α-SMA expression the results were also statistically highly significant when we compared the left side with the rectum. But unlike the results of the ERG expression, the association with the tumor grade revealed significant correlations when we compared the MVD in well-differentiated tumors with moderate differentiation in both inside and outside the tumor vascularity. When the tumor was in T3 or T4 the mean of the MVD was statistically low compared with T1 or T2 around the tumor. Equivalent to the results of the ERG, no correlation could be detected with other variables like age, sex, LN involvement or survival rate (Table 6).

**Table 6:** Association of the clinicopathological features with MVD by α-SMA expression in tumor and adjacent areas among patients with CRC. MVD: microvascular density, α-SMA: alpha smooth muscle actin, CRC: colorectal cancer, n: number, SD: standard deviation, T: tumor; T1: tumor invades submucosa (through the muscularis mucosa but not into the muscularis propria); T2: tumor invades muscularis propria; T3: tumor invades through the muscularis propria into the pericolorectal tissues; T4: tumor invades through the visceral peritoneum or adheres to other adjacent organs or structures, LN: lymph node; N1: metastasis in one to thee regional lymph nodes; N2: metastasis in four or more regional lymph nodes. P-value <0.05 was considered significant.

Clinicopathological features (n = 48)	MVD by α-SMA expression			
	In tumor Mean (SD)	P and pairwise comparisons	Adjacent areas Mean (SD)	P and pairwise comparisons
Age				
<60	33.70 (15.89)	0.2289	27.32 (13.44)	0.0889
≥60	28.08 (15.19)		21.32 (9.41)	
Sex				
Male	25.54 (11.84)	0.0515	25.37 (14.05)	0.6615
Female	33.78 (15.43)		23.67 (10.30)	
Tumor location			0.0106	
Left	27.87 (10.58)	0.4680	36.44 (16.90)	left side vs. Rectum (P=0.0077)
Right	28.86 (19.16)		26.71 (20.35)	left side vs. right side (P=0.1933)
Rectum	34.24 (16.21)		20.06 (5.62)	right side vs. Rectum (P=0.4359)
Tumor grade		0.0055	0.0084	
I “Well differentiated”	69.0 (43.84)	Well vs. Moderate (P=0.0040)	45.0 (12.73)	Well vs. Poor (P=0.0554)
II “Moderately differentiated”	29.19 (15.00)	Well vs. Poor (P=0.1066)	22.10 (9.62)	Well vs. Moderate (P=0.0060)
III “Poorly differentiated”	35.5 (6.36)	Moderate vs. Poor (P=0.8518)	22.0 (5.66)	Moderate vs. Poor (P=0.9999
Tumor (T)				
T1+ T2	38.9 (23.71)	0.1531	32.9 (17.55)	0.0155
T3+ T4	29.69 (15.68)		21.72 (10.13)	
LN involvement				
Not involved	31.74 (16.34)	0.1356	31.0 (17.71)	0.1124
Involved				
N1	24.29 (10.51)		22.93 (12.82)	
N2	37.11 (18.19)		20.33 (6.0)	

Similar to the results reported by the ERG expression the mean MVD detected by the α-SMA shows no correlation between the MVD and the special histopathological variables (Table 7).

**Table 7:** Association of special histopathological variables with MVD by α-SMA expression in tumor and adjacent areas among patients with CRC. MVD: microvascular density, α-SMA: alpha smooth muscle actin, CRC: colorectal cancer, n: number, SD: standard deviation. P-value <0.05 was considered significant.

Special histopathological variables (n = 48)	MVD by α-SMA expression			
	In tumor Mean (SD)	P and pairwise comparisons	Adjacent areas Mean (SD)	P and pairwise comparisons
Inflammatory reaction				
Mild	41.29 (26.29	0.2569	27.71 (14.64)	0.0566
Moderate	28.33 (15.09)		32.42 (20.51)	
Severe	31.50 (15.87)		20.40 (7.56)	
Tumor budding				
low	29.92 (12.15)	0.9840	28.27 (11.50)	0.1729
Intermediate	30.43 (16.20)		19.25 (7.56)	
High	30.95 (17.58)		24.29 (12.92)	
Tumor deposits				
Not present	29.41 (14.01)	0.7681	24.97 (13.31)	0.7027
Present	31.33 (19.67)		22.83 (6.52)	
Lymphovascular invasion				
Negative	30.75 (17.01)	0.9187	26.76 (12.72)	0.0601
Positive	30.27 (14.24)		20.16 (8.85)	

The mean of the pericytes around the microvessels was confirmed by α-SMA expression. Unlike the results of MVD which show no significant correlation with the special histopathological features, the mean pericytes showed multiple significant associations, as seen in Table 8. First of all, when the tumor inflammation was severe, the mean number of pericytes in areas around the tumor was low when compared to both mild and moderate inflammation (P = 0.0108). Similarly, when the tumor deposit was positive, the mean number of pericytes of the vascularity outside the tumor was significantly low (P value 0.0086). Furthermore, when the lymphovascular invasion was not detected the mean number of pericytes was highly significant in the vascularity outside the tumor. On the other hand, no correlation could be seen between the mean of the pericytes and tumor budding.

**Table 8:** Association of special histopathological variables with mean pericytes α-SMA positive in tumor and adjacent areas among patients with CRC. MVD: microvascular density, α-SMA: alpha smooth muscle actin, CRC: colorectal cancer, n: number, SD: standard deviation. P-value <0.05 was considered significant

Special histopathological variables (n=48)	Mean pericytes α-SMA positive			
	In tumor Mean (SD)	P	In adjacent areas Mean (SD)	P and pairwise comparisons
Inflammatory reaction				0.0108
Mild	96.33 (2.25)	0.0502	97.71 (2.69)	severe vs. Mild (P = 0.0603), severe vs. Moderate (P = 0.0208)
Moderate	92.53 (6.95)		97.22 (2.62)	
Severe	89.45 (6.31)		93.86 (4.73)	
Tumor budding				
Low	93.27 (6.87)	0.3233	97.08 (3.70)	0.5402
Intermediate	92.64 (4.73)		95.57 (3.63)	
High	90.05 (7.30)		96.16 (3.15)	
Tumor deposits				
Not present	91.98 (6.51)	0.8473	96.34 (3.60)	0.0086
Present	91.40 (3.13)		91.83 (4.83)	
Lymphovascular invasion				
Negative	92.56 (7.14)	0.4467	97.92 (2.04)	0.0001
Positive	91.14 (4.93)		93.77 (4.29)	

## Discussion

As the second-most deadly cancer globally and a rising cancer in developing regions [18-19], CRC is not accompanied by a commensurate increase in research efforts in developing countries. In the last decades, the microenvironment of tumors and adjacent tissue in human cancers, including the colon, has been proposed as a crucial factor in the progress of tumors and in cancer therapy [2,6,14]. In earlier times, it was believed that cancer cells releasing proangiogenic molecules acted as the main mediators of tumor angiogenesis. Nowadays, the accumulative data points out the stromal cells in TME as the stimulator for angiogenesis in various cancers, including CRC, subsequently affecting other aspects of the cancer [7,20]. In this study, the general variables of CRC were initially analyzed. Several clinicopathological features were similar to those reported around the world; the males were affected more than the females, and most of the CRC patients presented as grade II and in T3. On the other hand, the age was lower than that reported by the United States (the median age was 63 years for rectal cancer and 69 years for colonic cancer) [19]. However, a significant increase in USA incidence was reported in patients younger than 50, and for this reason, a new recommendation has been established by the American College of Gastroenterology in 2021 to carry out screening for this cancer at the age of 45 [21]. 

The results showed that the highest mean of MVD was in the tumor areas, and the lowest was in the adjacent areas. On the contrary, and in a completely opposite way, the mean of pericytes positive for α-SMA was lower within the vascularity of the tumor itself than in the vessels of the adjacent tissue. In this concept, De Palma et al. found that the lack of stable interaction between pericyte and endothelial cells in cancer inhibits angiogenic sprouting, leading to vascular dysfunction associated with endothelial proliferation and vascular leakage. Therefore, they suggested that the pericytes may provide crucial stimuli for angiogenesis [7].

Gao et al. also found higher MVD in tumor regions than normal mucosa [22]. In contradiction to this, in their study, Raghuram et al. examined cases of CRC for MVD in tumors as well as adjacent normal areas, and unlike our results, they found that the MVD was lower in tumors than in adjacent normal areas [23]. However, the MVD in their results was correlated with lower grade and stage; similarly, our results show a significant correlation between increased MVD in well-differentiated tumor grades and in T1 and T2, both inside and outside the tumor. Furthermore, the results suggested that other variables may also affect the microvascular environment; among these variables was position. The mean of the MVD around the tumor in left-sided CRC cases was significantly higher than that of the right and rectum sides. A similar result of significantly high MVD in left-sided CRC was reported previously within the tumor [24], and a possible explanation was that the vascular endothelial growth factor (the mediator of angiogenesis that promotes endothelial cell migration and proliferation), which is secreted by tumor cells and could be present in a minimal amount in normal mucosal tissue, was stronger and expressed more in the left-sided and rectum regions of CRC [25].

The effects of the TME, particularly the angiogenesis and their markers, vary in different CRC TNM stages. Furthermore, the sensitivities of certain markers are higher in early stages [26]. In agreement with this concept, the mean of the MVD in this study was significantly high in early stages with tumor size T1 or T2, both inside and outside the tumor, but no correlation was detected when the tumor was in T3 or T4 and when there was LN involvement. A number of authors demonstrated no connection between the MVD value and the clinicopathological factors [18,22,27], nevertheless, many others have shown a strong association between the two [23,28]. This study showed that the percentage of patients with high tumor budding was more than those with intermediate and low tumor budding. However, there was no significant relation between the mean of MVD and the pericyte expression with tumor budding. There is uncertainty between the interaction of the microenvironment and tumor buds in different types of malignancies [29].

Unlike the correlation of MVD, the mean pericytes showed significant correlation with negative vascular invasion, negative deposits and adverse correlation with severe inflammatory reaction. These significant correlations were detected in areas adjacent to the tumor. In agreement with these results, Bose et al. suggested that the pericytes are more loosely connected to the vasculature in tumors than in normal tissues, they may be present around tumor vessels, with coverage ranging from 50% to 95% in carcinomas and melanoma [9].

Pericyte was recognized as one of the primary sources of cancer-associated fibroblast in malignancies and fibrosis. Hosaka et al. revealed that PDGF-BBPDGFRb signaling can cause pericytes to change to fibroblasts, and that the detachment of pericytes from the tumor's microvasculature can cause them to transdifferentiate into fibroblasts that greatly aided in tumor invasion and dissemination [10]. This finding may explain the significant low pericytes found in our results within the tumor. Nevertheless, there is increasing evidence that pericytes, which are strategically located between the bloodstream and the interstitial space, play a significant role in inflammatory processes [30].

Limitations

Due to the relatively small sample size the statistical data needs more investigations and expansion to confirm the relationships between the MVD and mean pericytes number with several histopathological features. Sample size limitations should be taken into consideration for future research. The cases were chosen from 2013 to 2015, due to the predetermined intention to complete the study, assess the survival of the patients and correlate the results with those of the current study. 

## Conclusions

The significant decrease in the mean pericyte number in the adjacent tissue of cancer with the presence of lymphovascular invasion, tumor deposit, and severe inflammation may indicate that the microenvironment outside the tumor might play a fundamental role in the progress of cancer, in which the pericyte participates as a principal player in this communication and progression. These findings may aid the suggestion that defective or transformed pericytes around the tumor can be implicated in the development of the cancer and, subsequently, the invasion and dissemination. Furthermore, since the pericytes are located between the bloodstream and the interstitial space, they may interact with the mediators of inflammatory processes. However, no association has been found between MVD and several clinical and pathological variables, such as age, sex, inflammatory reactions, tumor budding, lymphovascular invasion, and tumor deposits.
